# Research On Maize Disease Identification Methods In Complex Environments Based On Cascade Networks And Two-Stage Transfer Learning

**DOI:** 10.1038/s41598-022-23484-3

**Published:** 2022-11-07

**Authors:** Hongxin Liu, Haichen Lv, Jiajun Li, Yongshuo Liu, Limiao Deng

**Affiliations:** School of Science and Information Science, Qingdao Agricultural University, Qingdao, 266109 China

**Keywords:** Computer science, Image processing

## Abstract

Achieving accurate and reliable maize disease identification in complex environments is a huge challenge. This is because disease images obtained from natural environments are often in complex contexts that may contain elements similar to disease characteristics or symptoms. Based on cascade network and two-stage transformation learning, the new method is proposed in this paper and applied the improved method to the task of identification and classification of four maize leaf types in a complex environment. The proposed method has a cascade structure which consists of a Faster R-CNN leaf detector (denoted as LS-RCNN) and a CNN disease classifier, named CENet(Complex Environment Network). The LS-RCNN detector with an attention mechanism was used to detect maize leaves from the image, and the CENet model further classified the leaf images detected in the first stage into four categories: Cercospora leaf spot, Common rust, Northern Leaf Blight, and Healthy, which allowed image features to be extracted more efficiently. The subsequent use of a two-stage transfer learning strategy to train CENet models of disease images in complex contexts allows for faster training of the models while ensuring accuracy. The experimental results show that the proposed method is used to identify four types of maize leaves with an F1-score of 99.70%, which is better than some popular CNN models and others' methods, and has a more obvious advantage in terms of training speed. The model proposed in this experiment has a positive significance for exploring other Crop variety identification and classification under complex backgrounds.

## Introduction

Maize is a major crop in China, with the largest planting area and yield, and also plays an important role in light industry, animal husbandry, and the national economy. Maize diseases not only reduce the maize yield but also affect the development of related industries and economies. At present, the manual method is the main method to identify maize diseases in China. The labor process of using manpower to identify maize diseases is not only inefficient, but also easy to be disturbed by subjective factors such as fatigue and emotion, and can only be identified when the obvious symptoms appear^1^. Therefore, how recognizing disease of maize leaves quickly and accurately and taking appropriate control measures is of great significance to ensure maize production.

The research on crop image disease recognition abroad began in the 1980s. Researchers have extensively used a variety of traditional machine learning methods to study the image recognition technology of agricultural diseases, including the support vector machine classifier method^2^, PNN method^3^, K-nearest neighbor classification method^4^, BP network method^5^, and so on, which has played a positive role in promoting the application of information technology in agricultural disease image recognition research. However, the traditional machine learning method has some shortcomings, such as limited learning and expression ability, manual extraction of features, and unsuitable for processing large amounts of data.

The deep learning method can effectively solve the problem of big data learning and modeling. In recent years, researchers have carried out a lot of research work in agricultural disease image recognition based on deep learning. Chen et al.^6^ proposed a new network called SE-MobileNet, which achieved an average accuracy of 99.78% and showed the feasibility and effectiveness of the deep learning network. Hammad Saleem et al.^7^ proposed an image-based deep learning meta-structure model to identify plant diseases. Long et al.^8^ proposed a recognition method based on a convolutional neural network and transfer learning for Camellia oleifera disease image recognition, and the average recognition accuracy reached 96.53%. Based on the characteristics of maize foliar diseases, Zhao et al.^9^ applied the threshold method, area marker method, and Freeman link code method to diagnose five major diseases of maize foliage with an accuracy of more than 80%. Liu et al.^10^ applied the Triplet loss double convolution neural network structure to study the features of corn images and then used the SIFT algorithm to extract texture features, and the classification accuracy was above 90%. Zeng and Li^11^ proposed the Self-Attention Convolutional Neural Network (SACNN) to identify crop diseases, and extensive experimental results showed that the recognition accuracy of SACNN on AES-CD9214 and MK-D2 was 95.33% and 98.0%, respectively. Chen, et al.^12^ proposed a new method to automatically detect and classify plant leaf diseases based on image processing techniques, which could effectively identify whether a plant was a pest or disease plant. Compared with the traditional machine learning methods, a deep learning framework can automatically learn the features contained in the image data. When the data set reaches a certain size, it can achieve better accuracy and robustness in the agricultural disease image recognition task. However, the application of deep learning in agricultural disease image recognition still has some problems, such as large training data set, over-reliance on data annotation, limited generalization ability of the model, and high requirements on hardware computing power.

Deep transfer learning method can use the learned knowledge in the field of big data to assist in the building data model in the field of smaller goals, directly reducing the size of the target domain modeling for data requirements, which includes the research field of agricultural disease image recognition. Researchers have carried out some related research work^13–15^, which used some existing large image datasets to assist in establishing the image recognition model of target disease with small sample data, and achieved certain results. Chen, Zhang, et al.^16,17^ proposed models generated by transfer learning for identifying plants and showed good results, demonstrating that the models trained on the public dataset still had good detection performance in complex environments. Moreover, the use of transfer learning in experiments can also reduce the data size requirement for modeling.

Although deep learning models for agricultural disease recognition are becoming more and more mature and some research results have been achieved, however, most of the research is based on disease images collected in the laboratory environment, and few studies focused on disease recognition in the actual farmland environment. When these methods are applied to the actual farmland environment, the detection and recognition results are easily affected by the complex environment and the image shooting environment. The recognition accuracy will be greatly reduced, and the applicability is poor with limitations. How to accurately recognize maize diseases in complex environments is still a great challenge. To solve this issue, the main contributions and novelty of this paper are as follows:

- We proposed an effective cascade network for maize disease identification in complex environments, which were composed of a Faster R-CNN leaf detector (denoted as LS-RCNN) and a CNN disease classifier (denoted as CENet).
- Two-stage transfer learning strategy was proposed to successfully train the disease classifier CENet, which allowed the model to converge faster, and be more suitable for disease recognition in the natural environment.
- Due to the lack of public data sets available on maize diseases in the natural environment, we constructed a maize disease dataset which contained 3842 laboratory images from Plant Village and 3380 natural images taken in field conditions.

The rest of this paper is organized as follows. In “Materials and methods” section, we elaborate on the proposed model and introduced the model structure in detail. “Results” section provides experimental results and analyses of our datasets. Then, discussions are given in “Discussion” section. Finally, we give conclusions and directions for future works in “Conclusion” section.

## Materials and methods

### Materials

#### Data collection

In this experiment, corresponding datasets were created for different types of maize leaves, which can be accessed at https://github.com/gitergo/Datasets/. Images in the lab dataset were obtained from Plant Village^18^, an open-access repository containing pest and disease images of many crops that have been used by many scholars with good results. Therefore, we selected four types of maize leaf images from Plant Village to form the laboratory dataset, which has a relatively simple background and is easy to identify and can be contrasted with the complex images in the natural environment. Most of the images in the natural environment dataset were acquired through field photography in Qingdao. Due to the limited variety of maize leaves available from field photography, we downloaded some open-source data on the natural environment as a supplement. All experimental protocols complied with all relevant guidelines and regulations.

The four categories of corn leaves were Cercospora leaf spot, common rust, Northeast leaf blight, and Healthy. Table 1 shows the number of images collected for each category, the number for training, validation, and testing, and their total number.

**Table 1:** Details of the data set.

Datasets	Category	Training	Validation	Testing	Total
Laboratory	Cercospora leaf spot Common rust Northern Leaf Blight Healthy	360 834 691 817	103 235 187 219	51 118 105 122	514 1187 983 1158
Natural environment	Cercospora leaf spot Common rust Northern Leaf Blight Healthy	171 1052 358 785	49 300 102 225	25 150 51 112	245 1502 511 1122

**Figure 1:**
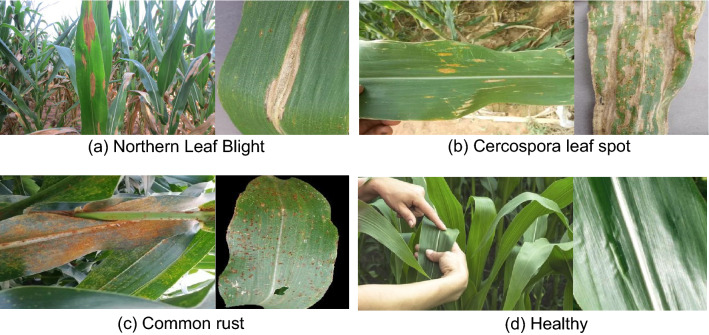
Sample images from natural environment datasets and laboratory datasets.

Figure 1 shows some sample images of the natural environment dataset and the laboratory dataset, as well as the differences in their backgrounds.

#### Data augmentation

We performed data enhancement on the existing image data (especially the natural environment) for data enhancement to achieve the purpose of increasing data volume, enriching data diversity, improving the generalization ability of the model, expanding the sample space, and reducing the influence of unbalanced data.

**Figure 2:**
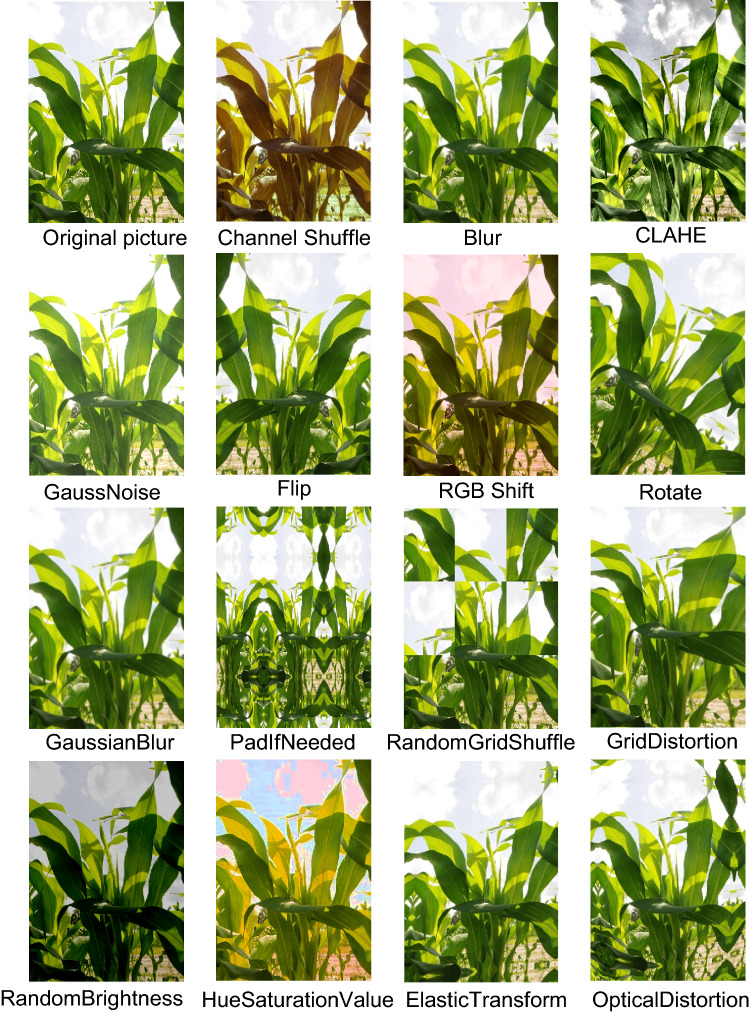
Data enhancement methods.

We used 15 data enhancement methods as shown in Fig. 2. These methods come from the OpenCV-based implementation of the Albumentations library^19^, a fast and flexible open-source library for image enhancement that provides many various image conversion operations. In most image conversion operations, Albumentations enhancement is faster than other commonly used image enhancement tools.

### The proposed method

**Figure 3:**
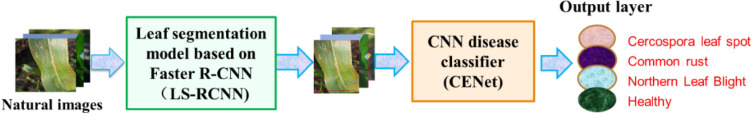
The proposed cascade networks.

**Figure 4:**
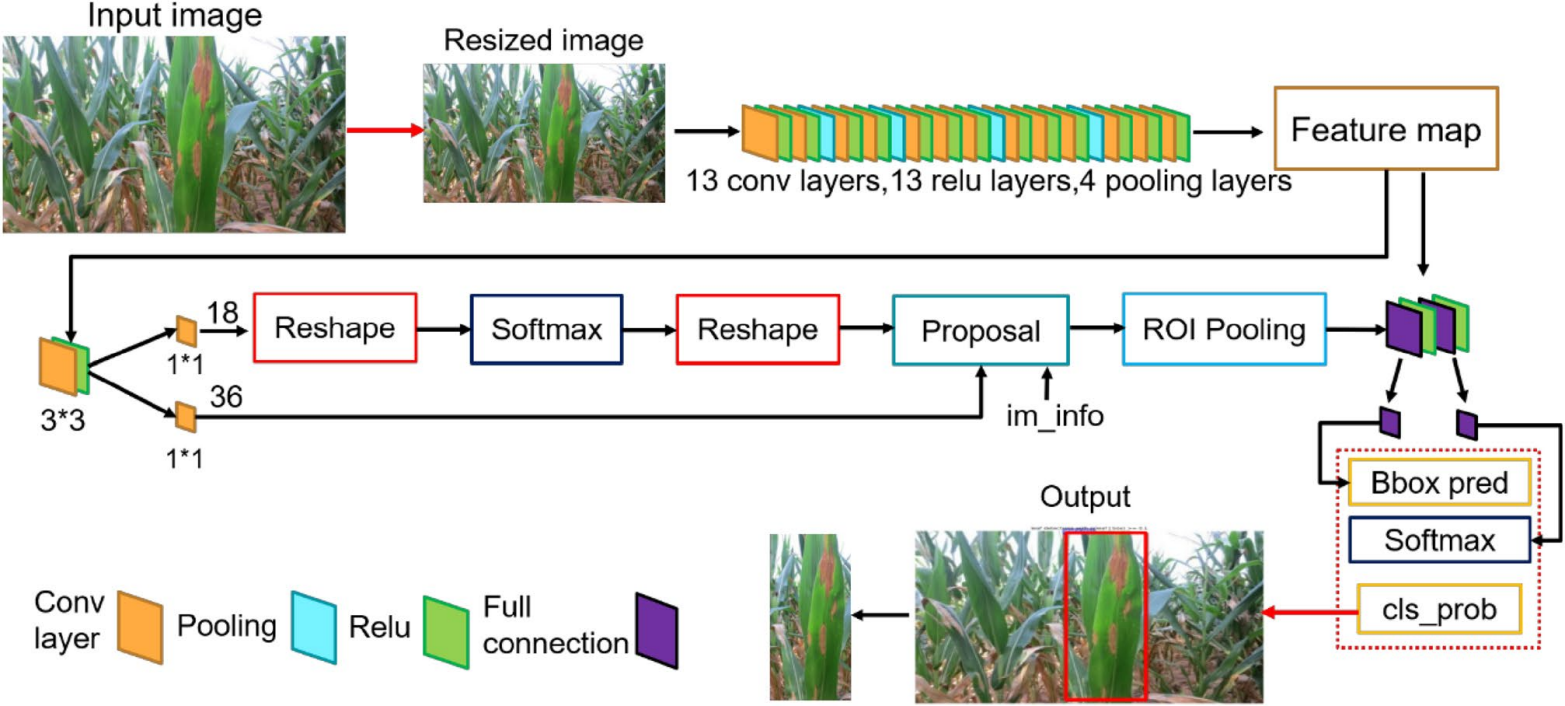
Structure of LS-RCNN model.

**Figure 5:**
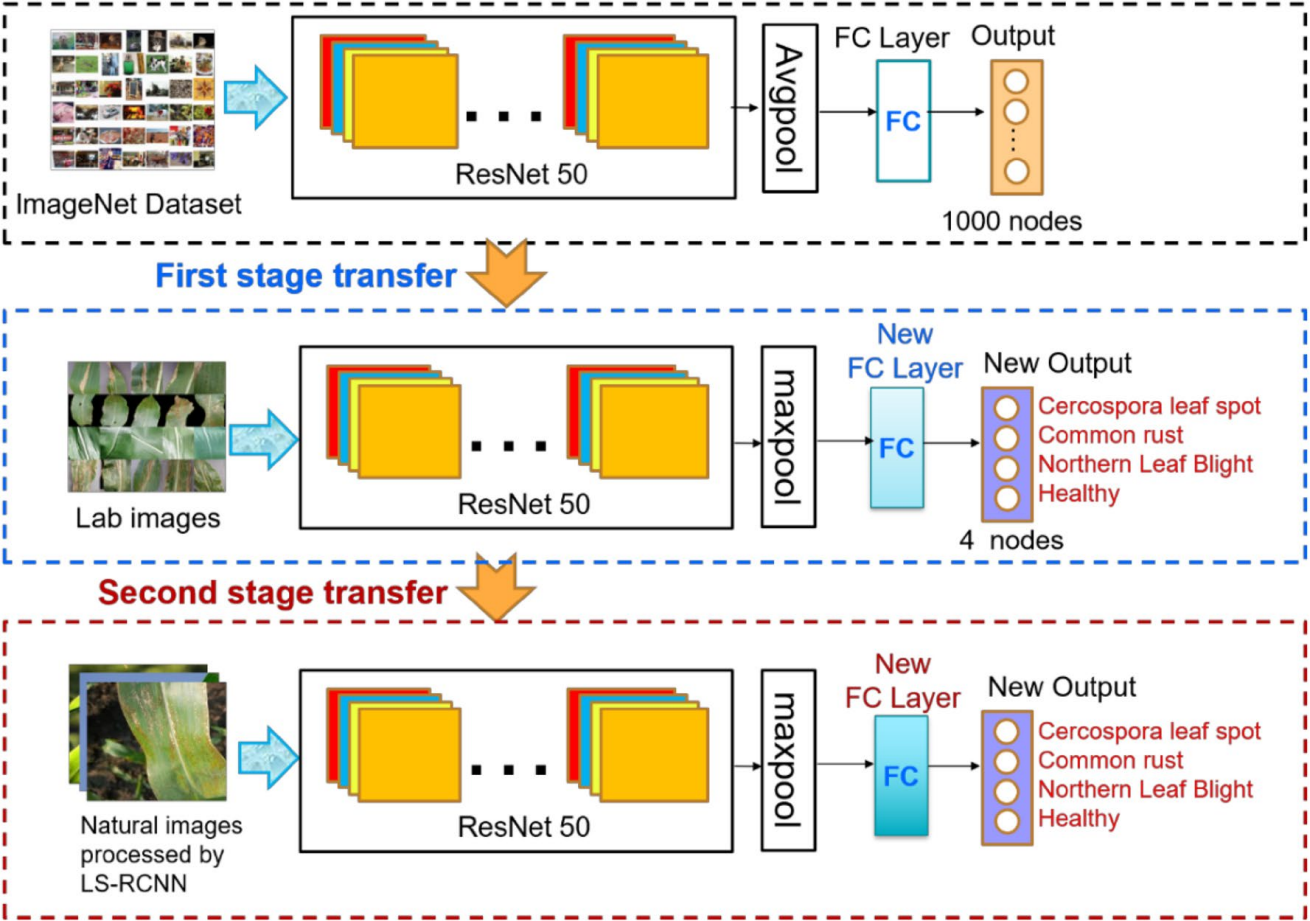
Architecture and training of CENet.

**Figure 6:**
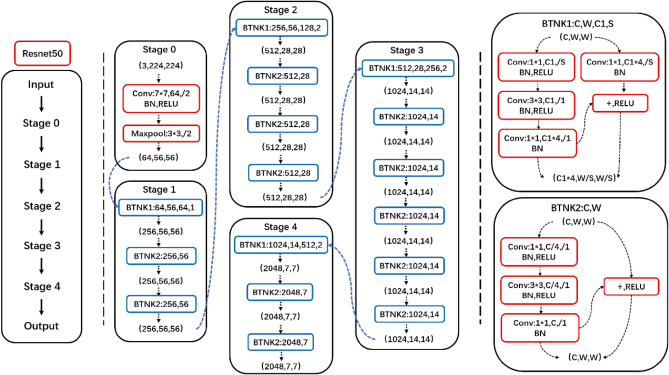
Resnet50 Architecture.

The proposed disease method had a cascade structure which consisted of a Faster R-CNN maize leaf detector (LS-RCNN) and a CNN leaf disease classifier (CENet), as shown in Fig. 3. First, disease images in the natural environment were input to the LS-RCNN to detect and separate the maize leaf from the complex background. Then the separated maize leaf was input into the trained CENet model to perform disease identification. Due to the complexity of the whole model, we first give a brief overall structure of the proposed cascade networks (Fig. 3) and then divided it into two parts depicted in detail in Figs. 4 and 5, and the structure of ResNet50 is described in detail in Fig. 6.

#### Leaf segmentation model based on Faster R-CNN (LS-RCNN)

Nanehkaran et al.^20^ proposed a detection method of image segmentation followed by image classification for plant disease leaves, and the detection results showed that most of the diseases were effectively detected under complex background conditions. To reduce the influence of complex background on recognition performance, we constructed the LS-RCNN model based on Faster R-CNN^21^ to extract the key regions of the maize leaf image from the background before they were fed into the CENet model for training and recognition. Figure 4 shows the model structure of LS-RCNN. Structurally, LS-RCNN had integrated feature extraction, proposal extraction, bounding box regression, and classification all into one network, which made its comprehensive performance improved, especially in the detection speed.

First, the LS-RCNN model used a basic set of conv + relu + pooling layers to extract feature maps of maize images, which were shared with the subsequent RPN and fully-connected layers. Then, the RPN network generated region proposals for the maize leaves, which used softmax to determine whether the anchors were positive or negative, and then used the bounding box regression to correct the anchors, eliminated those that were too small and out of bounds, and obtained the exact proposals for the maize leaf region. Next, the Roi Pooling layer collected the input feature maps and proposals and extracted the proposal feature maps after synthesizing the information, which was sent to the subsequent fully connected layer to determine the target class. At last, the category of the proposal was calculated by using the proposal feature maps and the final position of the detection box was obtained by bounding box regression to generate a detection box for the maize leaves. Thus, a new image was generated, which contained the detected maize leaf from each detection box.

#### CENet model based on two-stage transfer learning

To further solve the disease recognition problem in complex backgrounds, a two-stage transfer learning strategy was proposed to train an effective CNN deep learning model for disease images in complex backgrounds. Figure 5 shows the architecture and the training process of the CENet model for complex environments.

Since Alexnet^22^, the CNN structure has been continuously deepened. VGG^23^ and GoogLeNet^24^ have 19 and 22 convolution layers respectively. With the increase of network depth, the existence of gradient disappearance problems makes network training more difficult, and the convergence effect is poor, so ResNet is introduced. ResNet proposed by He et al.^25^ can effectively solve the deep network degradation problem. So, the ResNet50 model (Fig. 5) was used for transfer learning in this paper.

ResNet50 model was first pre-trained on the ImageNet dataset, and then the pre-trained model was trained by parameter transfer on the maize disease dataset obtained in the laboratory, which was the first stage of transfer learning. In the first-stage transfer learning, we replaced the average-pooling-based GlobalPool layer with a max-pooling layer and replaced the fully connected (FC) layer and classification layer with a new FC layer and classification layer. The new classification layer had four output nodes instead of 1000. Then the trained model was further transferred to the domain of natural images, which was the second stage of transfer learning. In the second-stage transfer learning, we replaced the FC layer and classification layer with a new FC layer and classification layer. Specifically, the region of interest was extracted by LS-RCNN to obtain the background simplified natural environment dataset and then was input into the ResNet50 model trained in the previous stage as training samples. In this way, the training process was completed and a well-trained CENet was obtained.

## Results

### Experimental setup

During training and testing, each image in the dataset is processed to fit the model, and the detailed image sizes are shown in Table 2. Hardware environment was CPU: Intel(R) Xeon(R) CPU E5-2678 v3 @ 2.50 GHz; GPU: NVIDIA GeForce RTX 2080 Ti; Number of floating point operations per second: 13.13 TFLOPS; Graphics Memory:11 GB; Motherboard Model: X10DRG-O + -CPU; Software environment was Mirror:Pytorch 1.8.1-Horovod;Mirror Description:Python3.7,CUDA 11.1, cuDNN 8.0.5, Pytorch 1.8.1, Horovod 0.22.1, Ubuntu 18.04, VNC, NVCC11.1, OpenMPI 4.0.0;

**Table 2:** Training parameters of models/detector.

Model / Detector	Training dataset	Transfer learning	Leaf detector	Learning rate	Momentum	Weight decay	Batch size	Input size	Step size	Epochs / Iters
[1]VGG16 [1]AlexNet [1]GoogleNet [1]ResNet18 [1]ResNet50 [1]Wide_ResNet50_v2 [1]ResNet101	Laboratory Laboratory Laboratory Laboratory Laboratory Laboratory Laboratory	/ / / / / / /	/ / / / / / /	0.001 0.001 0.001 0.001 0.001 0.001 0.001	0.9 0.9 0.9 0.9 0.9 0.9 0.9	/ / / / / / /	16 16 16 16 16 16 16	224 × 224 224 × 224 299 × 299 224 × 224 224 × 224 224 × 224 224 × 224	4 4 4 4 4 4 4	50 50 50 50 50 50 50
[2]ResNet50 [2]ResNet50	Laboratory Laboratory	one-stage two-stage	/ /	0.001 0.001	0.9 0.9	/ /	16 16	224 × 224 224 × 224	4 4	100 100
[3]ResNet50	Laboratory	/	/	0.001	0.9	/	16	224 × 224	4	50
[4]LS-RCNN [4]ResNet50 [4]ResNet50	Nature Nature Nature	/ / /	/ / LS-RCNN	0.001 0.001 0.001	0.9 0.9 0.9	0.0005 / /	256 16 16	375 × 500* 224 × 224 224 × 224	9000 4 4	15,000 100 100
[5]VGG16 [5]AlexNet [5]GoogleNet [5]GoogleNet* [5]OurModel	Nature Nature Nature Nature Nature	/ / / one-stage two-stage	/ / / / LS-RCNN	0.001 0.001 0.001 0.001 0.001	0.9 0.9 0.9 / 0.9	/ / / 1e-3 /	50 50 50 50 50	224 × 224 224 × 224 299 × 299 299 × 299 224 × 224	4 4 4 7 4	100 100 100 100 100

The experiment is divided into five parts. To ensure the fairness of the experiments, we used some hyperparameter settings in the comparison experiments. After many trials, we obtained the appropriate values of the model parameters. The hyperparameters of each part of the experiment are shown in Table 2, where [number] indicates which part of the experiment the model belongs to. In addition, 375 × 500* is the maximum input size supported by LS-RCNN, and GoogleNet* is the GoogleNet with the method proposed by Hu et al.^26^.

### Recognition performance comparison of different convolutional networks

To verify whether the introduction of ResNet50 has a better recognition effect, we set up a control experiment and introduce other mainstream CNN network structures into the model. The deeper layers of VGG16^23^ make the feature map wider and suitable for large datasets like the corn disease image dataset we built, while GoogleNet^24^ can ensure that the perceptual domain of each layer remains the same. With the deepening of the network, the network becomes more accurate, and the weight of the network can also be effectively reduced by using this structure. AlexNet^22^ adds a normalized LRN layer, which makes the accuracy higher. ResNet18^27^ is proposed to solve the problem of gradient disappearance or gradient explosion as the network becomes deeper and deeper. The experimental results of Wide_ResNet50 proposed by Zagoruyko & Komodakis^28^ show that the performance of the network can be improved by increasing the width, and the training efficiency of Wide ResNet is higher than that of the ResNet family for the same order of magnitude of parameters. ResNet101^25^ has a new residual unit, which makes training easier and improves generalization. By comparing ResNet50 with other CNN networks, the advantages and disadvantages of our corn disease recognition network can be effectively evaluated.

In the first part of the experiment, we continuously adjust the training hyperparameters, including learning rate, optimizer, and batch size, so that the model can obtain higher stability and complete the network training faster while obtaining higher accuracy, and the optimal hyperparameters are shown in Table 2. Finally, we identified ResNet50 as the optimal model and continued to optimize it so that it had better performance to recognize images with complex backgrounds. Detailed parameters are listed in Table 2 [1].

**Figure 7:**
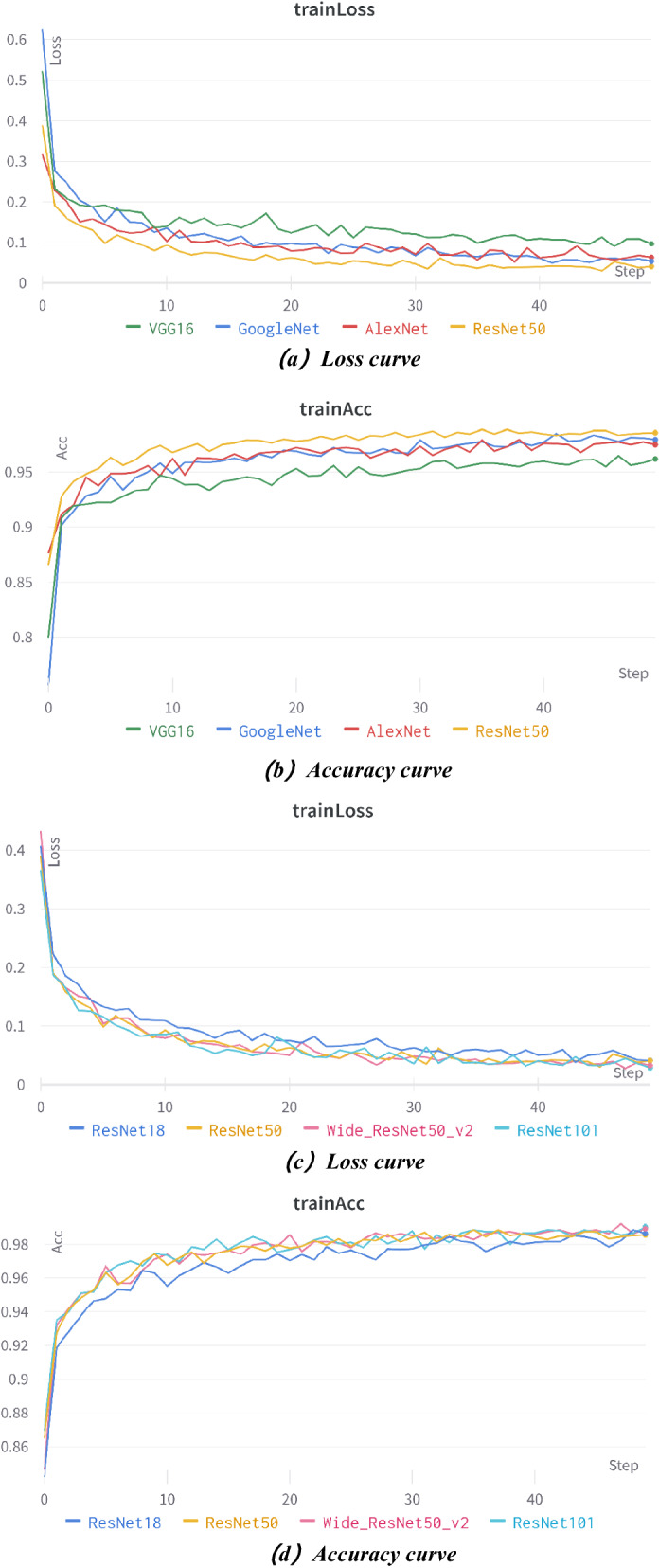
Comparison of recognition results among different convolutional networks.

The comparison of the loss rate of the network models with the number of training rounds after trained 50 epochs on the laboratory (public) dataset is shown in Fig. 7a and c, and the comparison of the recognition accuracy is shown in Fig. 7b and d.

Figure 7 shows that all the networks fit quickly in the first 2 epochs and the accuracy rate increases rapidly. Then the loss rate decreases slowly and the accuracy rate increases slowly in about 3–20 epochs, and then the loss rate tends to be stable and the accuracy rate also tends to be stable after 21 epochs, and the models begin to converge. Among the seven networks, Resnet50, wide_Resnet50_2, and Restnet101 have better recognition, excellent performance, and rapid convergence, with the highest accuracy of 98.52%, 98.66%, and 99.19%, respectively. The following are Resnet18, Alexnet, and GoogleNet with the highest accuracy of 98.25%, 98.25%, and 98.39%, respectively. And the highest accuracy of vgg16 is only 96.37%.

**Figure 8:**
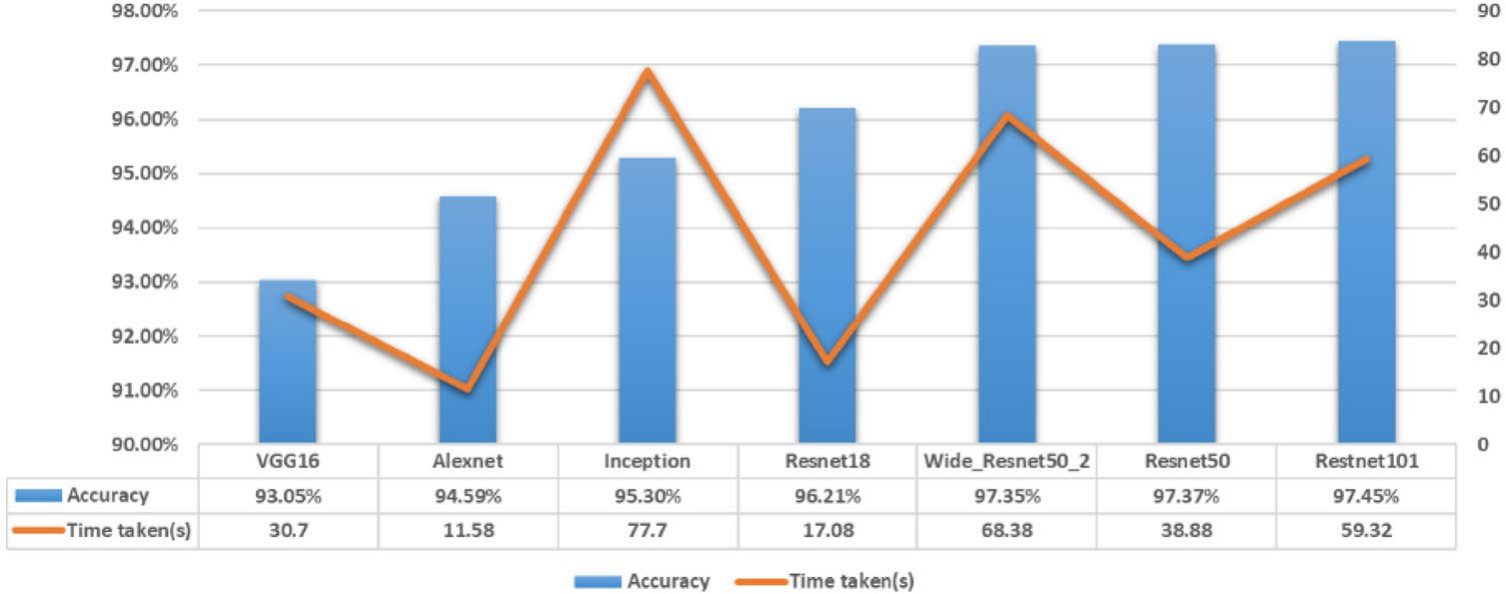
Time of training the model and the accuracy of the model.

The average training accuracy and consumed time after 50 epochs of training are shown in Fig. 8, in which the accuracy of each model is ranked in ascending order and the consumed time is also shown.

It can be found from Fig. 8 that the models with higher accuracy (e.g., Resnet50, Wide_Resnet50_2, Restnet101) usually take more time. Conversely, models with short time consumption do not have high recognition rates.

Therefore, making a tradeoff between the recognition accuracy and time spent during training, Resnet50 network demonstrated the best performance and was used for further optimization on datasets with complex backgrounds.

### Comparison between two-stage transfer learning and traditional transfer learning

In the second part of the experiment, we tested two-stage transfer learning against traditional transfer learning to demonstrate the feasibility and superiority of two-stage transfer learning. Detailed parameters are listed in Table 2 [2].

**Figure 9:**
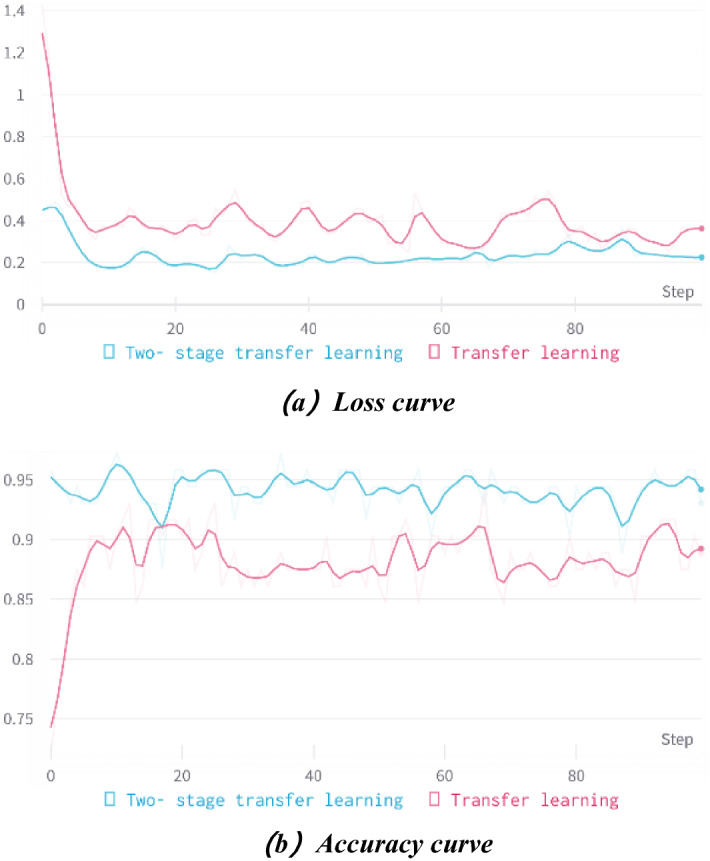
Comparison between traditional transfer learning and two-stage transfer learning.

Figure 9 shows the comparison of two-stage transfer learning with traditional transfer learning. Figure 9a is the loss curve, and Fig. 9b is the curve of recognition accuracy.

Figure 9 shows that both methods fit quickly in the first 4 epochs. Then the accuracy increases rapidly, and the loss rate slowly decreases and tends to be smooth in the subsequent epochs. Finally, the accuracy rate slowly increases and tends to be smooth, and the model converges. The accuracy of the two-stage transfer learning technique is higher, with the highest accuracy of 97.22% and the lowest loss rate of 0.1546; the accuracy of traditional transfer learning is relatively lower, with the highest accuracy of 93.06% and the lowest loss rate of 0.2501. The recognition effect of two-stage transfer learning is significantly better than that of traditional transfer learning.

### Recognition effect of different numbers of amplified images

In the third part of the experiment, we examined the relationship between accuracy and the number of training images and tested the effect of image amplification on recognition performance. Detailed parameters are listed in Table 2 [3].

**Figure 10:**
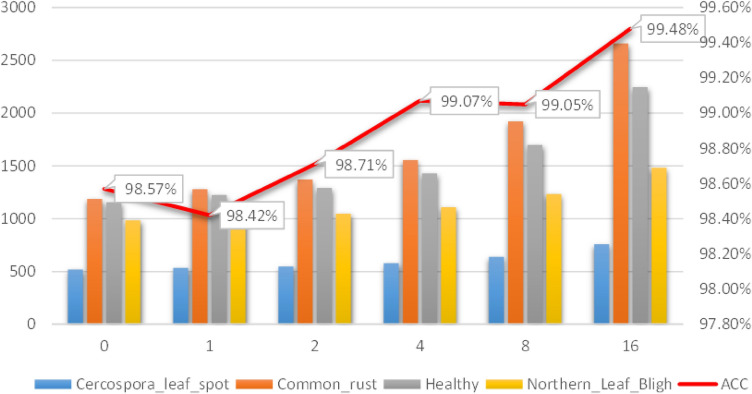
Change of accuracy when natural data sets are expanded exponentially by 2.

We conducted offline supervised data enhancement on the data set in the natural environment, and the accuracy change with the size of the amplified dataset is shown in Fig. 10.

Experimental results show that on the whole, the accuracy increases with the increase of the size of data sets, which indicates that the relationship between data size and accuracy is proportional, and the larger the data size, the higher the accuracy of the model is. However, when the data is amplified to 1 and 8 times, the accuracy does not increase, which indicates that data augmentation methods do not always have a positive impact on the accuracy. For example, some data augmentation methods such as CoarseDropout and RandomFog will reduce the accuracy of the model.

### Performance evaluation of LS-RCNN model

In the fourth part of the experiment, we trained LS-RCNN to remove the complex background of the leaves and obtained images of the natural environment with a simpler background. Meanwhile, we performed a control experiment to verify that this conjecture can indeed improve the recognition accuracy. Detailed parameters are listed in Table 2 [4].

**Figure 11:**
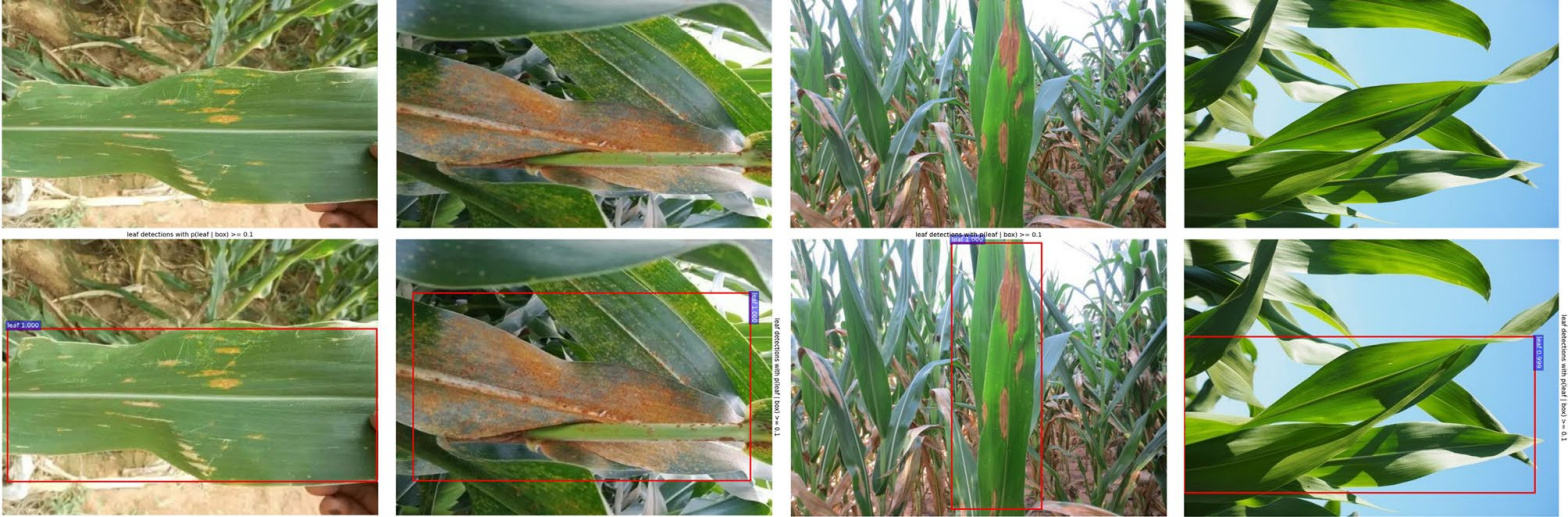
The effect of background segmentation using LS-RCNN.

Comparing the laboratory dataset with the natural dataset, we found that the background of the laboratory data was single, however, the background of the data in the natural environment was more complex and had interference features. Therefore, we used the LS-RCNN model to perform semi-supervised learning on the leaf as the region of interest, so that the natural data can achieve the purpose of separating the leaves from the background and reducing the interference factors of the complex background, as illustrated in Fig. 11.

**Figure 12:**
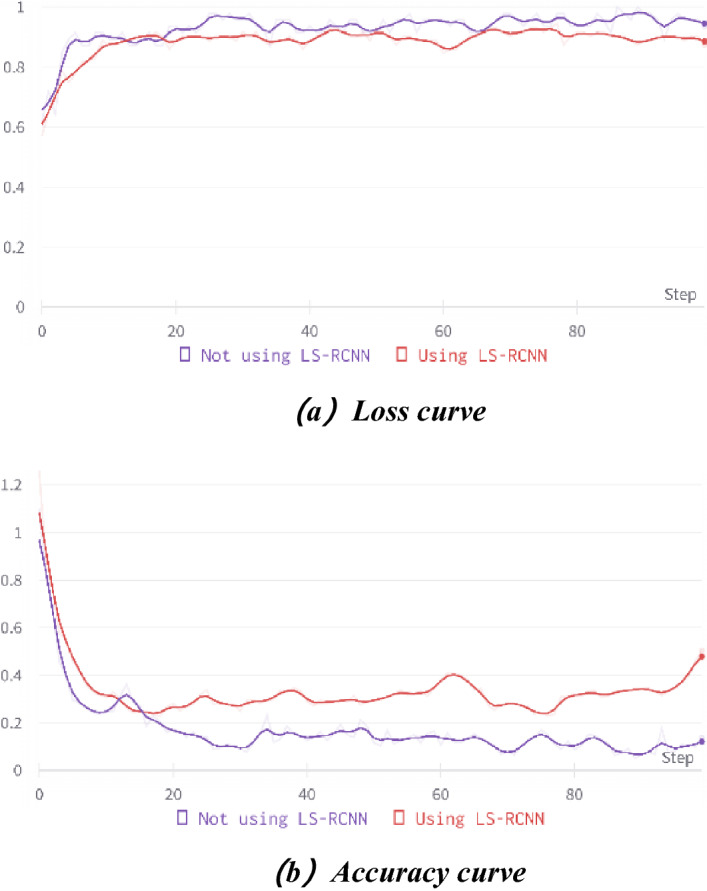
Recognition performance of LS-RCNN.

To evaluate the effect of leaf segmentation model LS-RCNN on the recognition performance, we performed experiments on two datasets: the original dataset with complex background and the dataset with complex background removed by LS-RCNN.100 epochs of training was performed on both datasets using the ResNet50 network, and the training loss curve is shown in Fig. 12a, and corresponding accuracy curve is shown in Fig. 12b.

Experimental results show that the two datasets fit quickly in the first 9 epochs and the accuracy increases rapidly; the loss rate decreases slowly and the accuracy increases slowly in about 10 to 26 epochs; after 27 epochs the loss rate leveled off and the accuracy leveled off, and the model converged. The accuracy of the dataset with complex background removed using LS-RCNN is higher, with the highest accuracy of 100% and the lowest loss rate of 0.06297; the accuracy of the original dataset is relatively lower, with the highest accuracy of 94.44% and the lowest loss rate of 0.2285.

### Performance evaluation of our method

**Figure 13:**
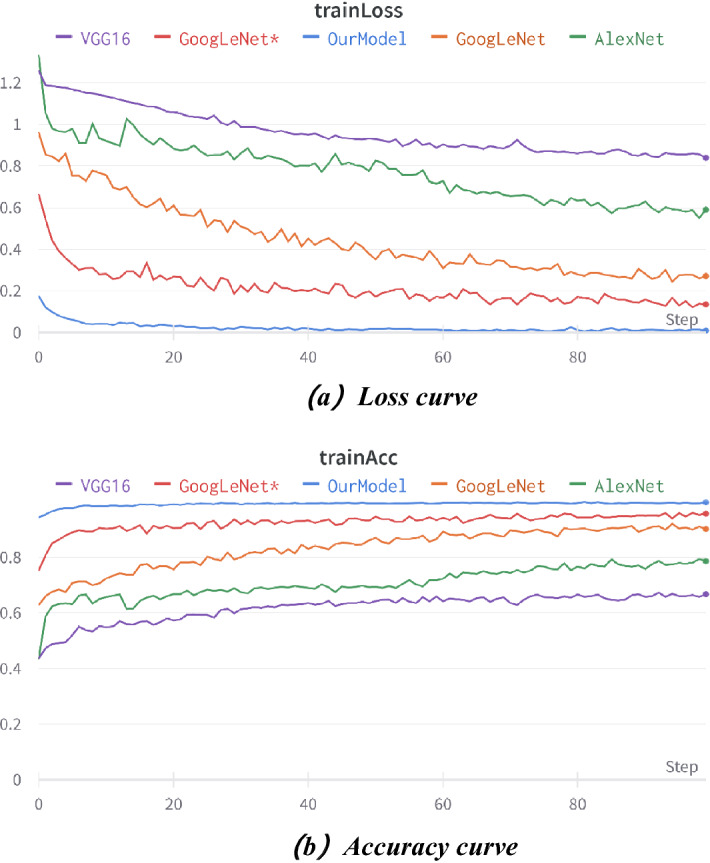
Comparison of recognition results of different convolutional networks for complex environment images.

In the fifth part of the experiment, to evaluate the performance of our proposed method, we conducted some experiments on the natural datasets. As there is no related research using the same data set, we tried to compare our method with some popular CNN models and some related methods ^26^(denoted as GoogleNet*) on our data set for a fair comparison. Figure 13 shows the comparison of our model with some related CNN models. Detailed parameters are listed in Table 2^5^.

As shown in Fig. 13, the loss curve of our model has converged to smooth after 20 iterations. The convergence trend of other models is not obvious, the fluctuation is larger and the loss value is higher within 100 iterations. Throughout the process, the accuracy of our model is higher than that of other models, and the fluctuation is smaller, which indicates that our model has higher detection performance and stable operation compared with the other models.

**Figure 14:**
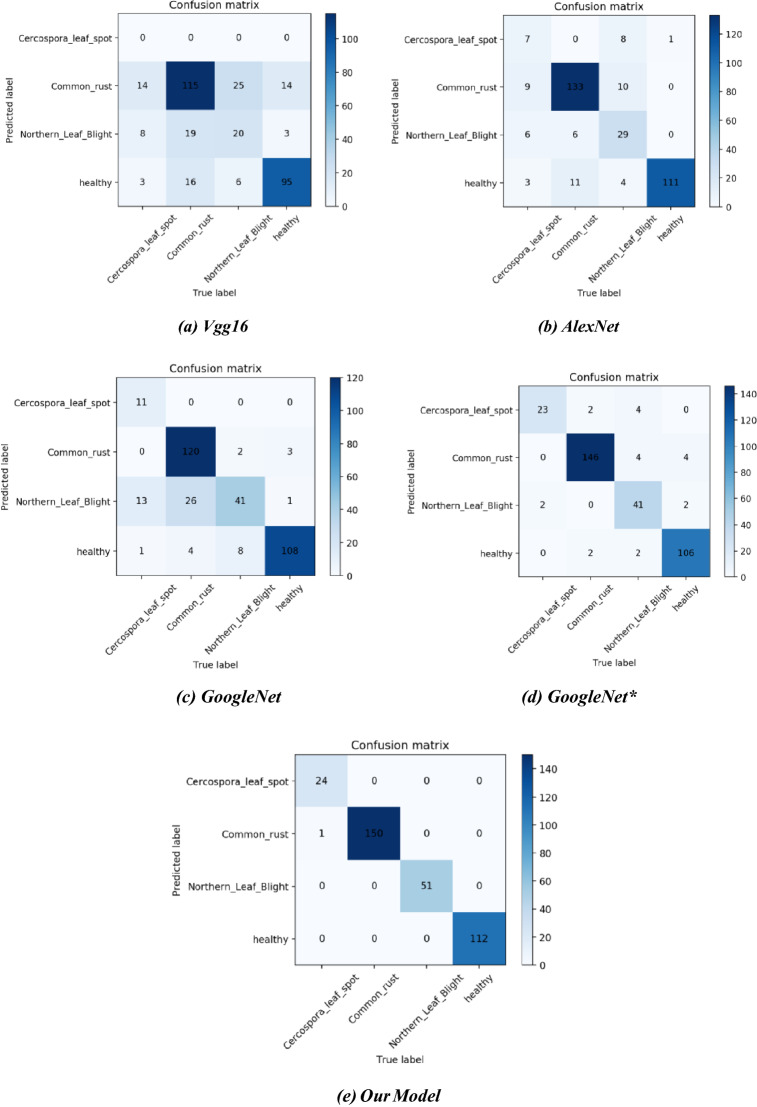
Confusion matrixes of the five models on the test dataset.

To further verify the recognition performance of the model, we performed testing experiments on the test set using the above five modes and plotted the classification confusion matrix based on the experimental results. as shown in Fig. 14.

Then, we calculated the accuracy, precision, recall rate, F1 score, Maro-F1, and Weighted-F1 of each category to evaluate the model performance, which is defined as follows:

1 $${\text{Accuracy }} = \frac{{{\text{TP}} + {\text{TN}}}}{{{\text{TP}} + {\text{TN}} + {\text{FP}} + {\text{FN}}}}$$

2 $${\text{Precision }} = \frac{{{\text{TP}}}}{{{\text{TP}} + {\text{FP}}}}$$

3 $${\text{Recall }} = \frac{{{\text{TP}}}}{{{\text{TP}} + {\text{FN}}}}$$

4 $${\text{F}}1 = \frac{{2 \times {\text{Precision }} \times {\text{Recall }}}}{{{\text{ Precision }} + {\text{Recall }}}}$$

5 $${\text{F}}1_{{\text{Macro }}} = 2 \cdot \frac{{{\text{ Precision }}_{{\text{Macro }}} \cdot {\text{ Recall }}_{{\text{Macro }}} }}{{{\text{ Precision }}_{{\text{Macro }}} + {\text{ Recall }}_{{\text{Macro }}} }}$$

6 $${\text{F}}_{{\text{Weighted }}} = \mathop \sum \limits_{{{\text{i}} - 1}}^{{\text{n}}} {\text{w}}^{{\left( {\text{i}} \right)}} {\text{F}}^{{\left( {\text{i}} \right)}}$$

where TP, TN, FP, and FN represent the number of true positives, true negatives, false positives, and false negatives, respectively, as defined in Table 3.

**Table 3:** Definition of TP \TN \FP\FN.

	Positive	Negative
True	True Positive(TP)	True Negative(TN)
False	False Positive(FP)	False Negative(FN)

Table 4 shows the model evaluation metrics of VGG16, AlexNet, GoogleNet, GoogleNet*, and Our Model for the maize leaf recognition tasks in complex environments. Table 4 shows that (since the recognition of VGG16 is not ideal and some values are not calculated, the models involved in the comparison are AlexNet, GoogleNet, GoogleNet*, and Our Model only) the average accuracy of our model is 99.70%, which is 6.21–16.86% higher than the other models. The average precision of our model is 99.83%, which is 9.43–27.82% higher than other models. The average recall of our model is 99.00%, which is 7.91–30.84% higher than other models. The average F1 score of our model is 99.41%, which is 8.87–30.06% higher than other models. The Weight-F1 of our model is 99.70%, which is 6.24–18.05% higher than other models. Our model showed excellent identification performance and outperformed the other models on all performance metrics. Table 5 shows that our model takes only a little more time than AlexNet, and has the highest recognition accuracy. This shows that under the same conditions, our model can perform image recognition in complex environments quickly, efficiently, and accurately.

**Table 4:** Comparison of our method with some related CNN models.

Model	Metric	Class				Average overall classes
		Cercospora leaf spot	Common rust	Northern leaf blight	Healthy	
VGG16	Accuracy (%)	68.05				
	Precision (%)	–	68.45	40.00	79.17	–
	Recall (%)	0.00	76.67	39.22	84.82	50.18
	F1 score (%)	–	72.33	39.60	81.90	–
	Weighted-F1 (%)	–				
AlexNet	Accuracy (%)	82.84				
	Precision (%)	43.75	87.50	70.73	86.05	72.01
	Recal l (%)	28.00	88.67	56.86	99.11	68.16
	F1 score (%)	34.15	88.08	63.04	92.12	69.35
	Weighted-F1 (%)	81.65				
GoogleNet	Accuracy (%)	82.84				
	Precision (%)	100.00	96.00	50.62	89.26	83.97
	Recall (%)	44.00	80.00	80.39	96.43	75.21
	F1 score (%)	61.11	87.27	62.12	92.70	75.80
	Weighted-F1 (%)	83.34				
GoogleNet*^26^	Accuracy (%)	93.49				
	Precision (%)	79.31	94.81	91.11	96.36	90.40
	Recall (%)	92.00	97.33	80.39	94.64	91.09
	F1 score (%)	85.19	96.05	85.42	95.50	90.54
	Weighted-F1 (%)	93.46				
OurModel	Accuracy (%)	99.70				
	Precision (%)	100.00	99.34	100.00	100.00	99.83
	Recall (%)	96.00	100.00	100.00	100.00	99.00
	F1 score (%)	97.96	99.67	100.00	100.00	99.41
	Weighted-F1 (%)	99.70				

GoogleNet* is the GoogleNet with the method proposed by Hu, R. et al.^26^.

**Table 5:** Comparison of completion time of the models.

Model	OurModel	VGG16	AlexNet	GoogleNet	GoogleNet*
Run Time (num_epoch = 100)	**37m33s**	**53m6s**	**38m16s**	**58m32s**	**58m7s**

## Discussion

### Solutions to low accuracy in complex environments

#### Two-stage transfer learning

The term transfer was first cited by Lorien Pratt in the field of machine learning. Pratt et al.^29^ proposed a new algorithm called Discriminability-Based Transfer (DBT), where the target network initialized by DBT learns significantly faster than the network initialized randomly. Chuong B Do and Andrew Ng^30^ explored the application of transfer learning in text classification. Schölkopf et al.^31^ proposed a method for learning a low-dimensional representation that is shared across a set of multiple related tasks. The proposed approach greatly improves the performance compared to learning each task independently. The application of transfer learning to Bayesian networks is discussed by Niculescu-Mizil and Caruana^32^ through transfer learning, the trained network model parameters are saved and reapplied in the new task, which makes the feature parameters of the original network model effectively used and increases the portability.

For the problem of low accuracy in natural scenes that occurs in the experiment, we proposed a two-stage transfer learning method to attempt to solve the problem of recognition accuracy caused by insufficient features of natural data and prevent overfitting problems.

We used the ResNet50 network as the base CNN architecture, set the first sample parameters as trained parameters on the ImageNet dataset, set the second sample parameters as trained parameters on a self-constructed natural environment dataset with a complex background, and used the two-stage transfer learning method to train the maize leaf disease image dataset. Experimental results demonstrated that the accuracy of two-stage transfer learning improved by 4.16% over traditional transfer learning, and had good performance in recognizing images with complex backgrounds in natural environments, which is an effective method to solve the low recognition rate of complex backgrounds.

#### Image segmentation based on Faster R-CNN

Faster R-CNN was used in the LS-RCNN model to separate maize leave from complex backgrounds for several main reasons: in recent years, Faster R-CNN has been widely used for image target recognition in agriculture^33^ because of its ability to automatically learn image features, and the Faster R-CNN is one of the most mature target detection algorithms; Faster R-CNN performs well on multiple datasets and is easy to transfer, and changes to the target classes in the dataset can be made to improve the detection speed. Faster R-CNN can integrate feature extraction, candidate region extraction, border regression, and classification into a single network, and use shared convolutional layers to improve detection speed.

For disease recognition in complex background, Li et al.^34^ improved Faster R-CNN for leaf disease detection in bitter melon in the field. Zeng and Li^11^ proposed a Self-Attention Convolutional Neural Network (SACNN), which extracts effective features of crop disease spots to identify crop diseases. Zhou et al.^35^ proposed a vegetable disease recognition model for complex backgrounds based on region proposal and progressive learning (PRP-Net). This model achieves an average recognition accuracy of 98.26%, which is 4.46 percentage points higher than that of the original region proposal network framework. So, we attempted to construct an LS-RCNN model based on Faster R-CNN to detect the regions of interest in natural images. LS-RCNN proved very effective for separating corn leaves from the complex environment and was very helpful to solve the problem of corn leaf disease identification in a complex environment.

### Limited number of images in complex environments

We found that recognition accuracy would be greatly affected by too few images in complex natural environments during two-stage transfer learning. To prevent possible overfitting problems with the limited dataset, we expanded the natural environment dataset in the following two ways: one was to download as many pictures as possible from the Internet, and the other was to use the data augmentation method.

Data enhancement is a common technique to increase the size and diversity of labeled training sets by using input transformations that retain the corresponding output labels. In computer vision, image enhancement has become a common routine technique to combat over-adaptation in deep learning models and is widely used to improve performance. While most deep learning frameworks implemented basic image transformations^36,37^, which were typically limited to certain variations of flipping, rotating, scaling, and cropping.

In addition, the speed of image processing in existing image enhancement libraries varies. In this paper, we used 15 data enhancement methods and amplified the dataset in complex environments by different orders of magnitude. Experimental results showed that, on the whole, data augmentation improved the recognition performance of the model, and solved the problem of limited data sets to a certain extent, as demonstrated in the previous research^38^. However, not all data enhancement methods are effective. Which method is more effective, or how much-amplified data is appropriate remains to be studied in the future.

## Conclusion

In this paper, we propose a new method based on cascade networks and two-stage transfer learning to identify maize leaf diseases in natural environments. Using our proposed method, the proposed model achieved an average accuracy of 99.70%, which is higher than most human experts and conventional neural network models. The proposed method not only eliminates the unnecessary feature extraction process but also improves the accuracy of disease recognition in complex backgrounds. The average F1-score of our method is 8.87–30.06% higher than other models in complex backgrounds and exceeds the prevailing deep learning methods. The proposed method provides a new and effective approach for maize seed retention disease identification in complex environments.

In the future, we will conduct research in two directions. First, we will try to integrate multiple region attention to model more complex fine-grained categories. Second, we will try to use a technique that is designed to be used to get more features by removing the complex background rather than focusing on the local area.

## Data Availability

The data that support the plots within this paper and other findings of this study are available from the corresponding author upon reasonable request.
